# Breaking the millisecond barrier on SpiNNaker: implementing asynchronous event-based plastic models with microsecond resolution

**DOI:** 10.3389/fnins.2015.00206

**Published:** 2015-06-08

**Authors:** Xavier Lagorce, Evangelos Stromatias, Francesco Galluppi, Luis A. Plana, Shih-Chii Liu, Steve B. Furber, Ryad B. Benosman

**Affiliations:** ^1^Equipe de Vision et Calcul Naturel, Centre National de la Recherche Scientifique UMR 7210, UMR S968 Inserm, Vision Institute, CHNO des Quinze-Vingts, Université Pierre et Marie CurieParis, France; ^2^Advanced Processors Technologies Research Group, School of Computer Science, University of ManchesterManchester, UK; ^3^Institute of Neuroinformatics, University of Zürich and ETH ZürichZürich, Switzerland

**Keywords:** SpiNNaker, neuromorphic, event-based models, microsecond, asynchronous, plasticity

## Abstract

Spike-based neuromorphic sensors such as retinas and cochleas, change the way in which the world is sampled. Instead of producing data sampled at a constant rate, these sensors output spikes that are asynchronous and event driven. The event-based nature of neuromorphic sensors implies a complete paradigm shift in current perception algorithms toward those that emphasize the importance of precise timing. The spikes produced by these sensors usually have a time resolution in the order of microseconds. This high temporal resolution is a crucial factor in learning tasks. It is also widely used in the field of biological neural networks. Sound localization for instance relies on detecting time lags between the two ears which, in the barn owl, reaches a temporal resolution of 5 μs. Current available neuromorphic computation platforms such as SpiNNaker often limit their users to a time resolution in the order of milliseconds that is not compatible with the asynchronous outputs of neuromorphic sensors. To overcome these limitations and allow for the exploration of new types of neuromorphic computing architectures, we introduce a novel software framework on the SpiNNaker platform. This framework allows for simulations of spiking networks and plasticity mechanisms using a completely asynchronous and event-based scheme running with a microsecond time resolution. Results on two example networks using this new implementation are presented.

## 1. Introduction

The ability of neurons to fire stereotypical action potentials with a very high temporal resolution, observed both *in vivo* (Chang et al., 2000; Tetko and Villa, 2001) or *in vitro* (Mao et al., 2001), points at the importance of temporal precision in neural coding. Bair and Koch (1996) analyzed the temporal properties of neurons in the medial temporal (MT) area of monkeys by using single-cell recordings. They demonstrated that 80% of the cells in area MT are capable of responding with a jitter of less than 10 ms, while the most precise cells have a jitter of less than 2 ms. At the sensory end, ganglion cells are known to emit action potentials with a time resolution in the range of milliseconds (Berry et al., 1997; Gollisch and Meister, 2008). However, some neurons can encode signals which are even faster than their own dynamics. Softky (1994) suggests that dendritic trees can be responsible for high-temporal coincidence detection with time constants faster than the ones of their neural membrane. Gerstner et al. (1996) have addressed this apparent paradox by using as an example the auditory system of the barn owl. They can locate a target sound with a precision of a couple of degrees, corresponding to a resolution of 5 μs. They show how neurons, which have synaptic and membrane time constants orders of magnitude larger, can phase-lock and respond to signals arriving coherently in a short time window. Learning plays a crucial role in shaping such connectivity, and in tuning cells to preferential input phases. Plasticity also mediates cross-modal interactions to give rise to the precise sound localization system in the owl (Gutfreund et al., 2002; Knudsen, 2002), where visual and auditory inputs are combined together to shape the neural circuitry responsible of such great temporal precision.

As the extent of the precision needed by models of spiking neural networks is still a matter of debate, having neural platforms capable of rapidly acquiring and generating sensory data at high temporal resolution becomes a valuable asset for scientific research. While mixed-mode VLSI multi-neuron chips can support high temporal resolutions by processing continuous analog signals (Indiveri et al., 2006; Brink et al., 2013; Benjamin et al., 2014), time-stepped digital platforms are bounded by the operating frequency of the global clock (Furber and Temple, 2007; Merolla et al., 2014). In order to investigate questions about required temporal precision in neural networks, we introduce a novel programming framework for SpiNNaker (Furber et al., 2014), a digital parallel architecture oriented to the simulation of large scale models of neural tissue. The approach introduced in this work leverages the event-driven nature of the platform to perform simulations with increased temporal resolution. We introduce a new collection of tools (spike sources and monitors, neural and plasticity models) oriented to sub-millisecond event-driven simulations and characterize the temporal behavior of the platform at different levels.

The paper is organized as follows: Section 2 describes the hardware and software architecture of SpiNNaker and its current limitations. Section 3 introduces our novel programming framework and the components provided for sub-millisecond simulation. Section 4 reports the time characterization of the platform using a new method for measuring latencies. It also present results from two example network models (sound localization and learning of temporal patterns) which require sub-millisecond precision. The networks are implemented in real-time on a 48-chip SpiNNaker board using the novel set of tools presented in this work. Finally the Conclusion Section summarizes the key temporal aspects of the software and models introduced in this paper.

## 2. The SpiNNaker platform

This section describes the hardware and software aspects of the SpiNNaker platform and the current limitations of the software implementation related to time resolution.

### 2.1. Hardware

**Figure 1:**
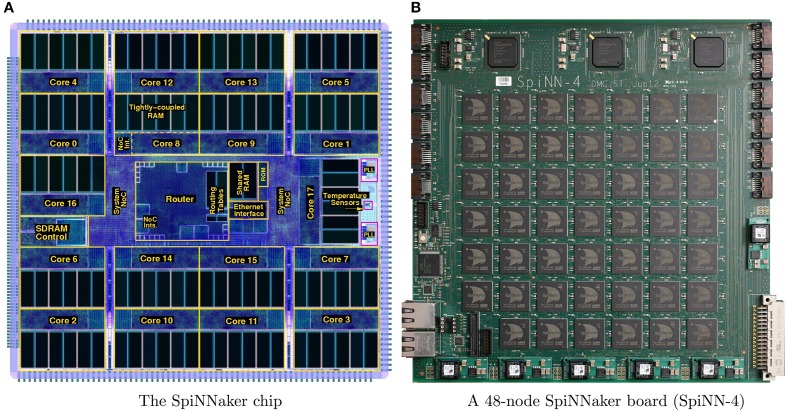
**System overview: (A) shows a plot of the SpiNNaker chip die, while (B) shows the largest prototype which consists of 48 SpiNNaker chips**.

The SpiNNaker chip is an application specific integrated circuit (ASIC) designed to realize large-scale simulations of heterogeneous models of spiking neural networks in biological real-time (Furber et al., 2014). Each SpiNNaker chip, Figure 1A, comprises 18 identical ARM968 cores each with its own local tightly-coupled memory (TCM) for storing data (64 kilobytes) and instructions (32 kilobytes). All cores have access to a shared off-die 128 megabytes SDRAM, where the relevant synaptic information is stored, through a self-timed system network-on-chip (NoC).

At the center of the chip lies a packet-switched multi-cast (MC) router (Wu and Furber, 2010) responsible for communicating the spikes to the local cores or to neighboring chips through 6 asynchronous bi-directional links. The router is capable of handling one-to-many communications efficiently, while its novel interconnection fabric allows it to cope with very large numbers of very small packets. Spikes are transmitted as 40 or 72 bit MC packets implementing the Address-Event Representation (AER) (Mahowald, 1994) scheme, where the information transmitted is the address of the firing neuron. Each packet consists of an 8 bit packet header, a 32 bit routing key identifying the neuron that fired and an optional 32 bit payload which is not normally used for neural applications (Wu and Furber, 2010). Every core within a SpiNNaker chip includes a communications controller which is responsible for generating and receiving packets to and from the router through an asynchronous communications NoC.

By combining multiple SpiNNaker chips together larger systems are formed. The SpiNNaker board with 48 chips (SpiNN-4), Figure 1B, is the largest prototype system available to-date and is currently being used as the building block for forming larger SpiNNaker machines. The SpiNN-4 board has 864 ARM9 cores, 768 of which can be used for neural applications, while 1 core per chip is dedicated for monitoring purposes and an additional one for fault-tolerant purposes (Furber et al., 2013). Additionally, there are 3 Xilinx Spartan-6 field programmable gate arrays (FPGA) chips that are used for inter-board communication purposes through the 6 high-speed SATA links. A previous study (Stromatias et al., 2013) demonstrated that a SpiNN-4 board is capable of handling up to a quarter of a million neurons (with millisecond update), with millions of current-based exponential synapses generating an activity of over a billion synaptic events per second, while each chip dissipating less than 1 Watt. The final SpiNNaker machine will utilize approximately 1000 SpiNN-4 boards and it aims at simulating a billion neurons with trillions of synapses in biological real-time.

### 2.2. Software

The SpiNNaker software can be divided into two parts, the software running on the chips and on the host. Each SpiNNaker chip runs an event-based Application Run-time Kernel (SARK) that has two threads, the scheduler and the dispatcher. The scheduler is responsible for queuing tasks based on a user-defined priority, while the dispatcher de-queues and executes them starting with the highest-priority task. Tasks with priorities set to minus one are pre-emptive, zero task priorities are non-queueable and are executed directly from the scheduler, while tasks with priorities set to one and above are queueable (Sharp et al., 2011).

The SpiNNaker application programming interface (API) is built on top of SARK and allows users to write sequential C code to describe event-based neuron and synapse models by assigning callback functions that respond to particular system events. Some example events are:

- **Timer Event:** A user-defined periodic event, usually set to 1 ms, which is used to solve the neural equations and update the synaptic currents.
- **Packet Received Event:** An event is triggered every time a core receives a spike (MC packet). It initiates a Direct Memory Access (DMA) transfer in order to fetch the pre-synaptic information from the SDRAM to the local memory. This DMA operation is autonomous, the ARM core may handle pending events or enter into a power-saving sleep mode.
- **DMA Done Event:** This event is generated by the DMA controller to inform the core that a DMA transfer has been completed. Each synaptic weight and conductance delay gets updated.

If there are no pending tasks the cores enter a low-power “sleep” mode.

On the host side PyNN (Davison et al., 2009), a high-level simulator-independent neural specification language, is used that allows users to described neural topologies and parameters using abstractions such as *populations* and *projections*. A tool named partition and configuration management (PACMAN) (Galluppi et al., 2012b) is responsible for mapping a PyNN description to a SpiNNaker machine based on the available resources, generating and uploading the relevant binary files, initiating a simulation and fetching the results to the host for further analysis.

### 2.3. Limitations of the current implementation

**Figure 2:**
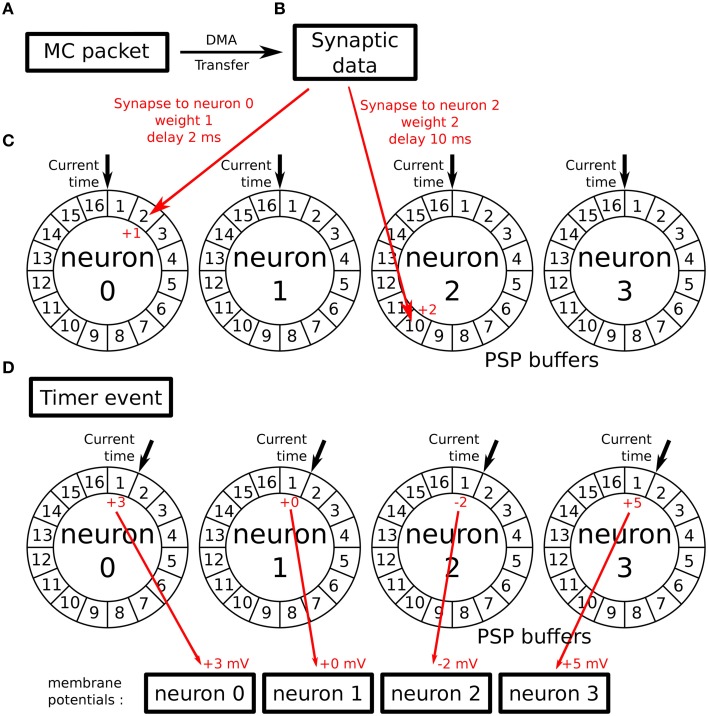
**Working principle of the PSP buffers: (A) a MC packet is received, carrying an incoming spike**. It triggers a DMA transfer. **(B)** As a result, the DMA Done event is triggered when the synaptic data associated with the spike is retrieved from SDRAM. **(C)** Synapses related to this spike are processed and their contributions are stored in the PSP buffers of the targeted neurons in the cell of the buffer corresponding to the synaptic delays. **(D)** When the next Timer Event is issued, the update process reads the contributions of previously received spikes for the current timestep and adds them to the neurons' membrane potential after updating its value (according to the used neural model).

In the SpiNNaker software available at the time of this work, neural models are implemented in a time-driven fashion. These models use the **Timer event** (see Section 2.2) to periodically update the state of the simulated neurons with a given timestep. Parallel to that update process, incoming spikes to the implemented neural population are processed through the **Packet Received Event** (see Section 2.2). This event looks up the different synaptic weights and delays relative to each connection. When the synaptic delay has been retrieved, the future contribution of the spike to the membrane potential of a given neuron is stored in its associated Post-Synaptic Potential buffer (PSP buffer). To implement the actual delay, the PSP buffers are ring buffers comprising one cell per simulation timestep. The periodic update process can then read this value at the timestep they need to be applied. This process is represented in Figure 2.

This implementation implies a trade-off between time resolution and memory usage. The memory space required by the PSP buffers is proportional to:

- the number of neurons simulated by the core (one buffer per neuron),
- the maximum synaptic delay allowed
- the resolution of the timestep

The standard implementation uses a maximum delay of 16 ms with a time resolution of 1 ms to simulate 100 neurons per core.

These values have been chosen taking into account spike propagation delays and the memory footprints of the synaptic buffers. Spike propagation in large SpiNNaker machines is guaranteed to happen within 1 ms. Original time-driven neural models on SpiNNaker therefore have a 1 ms time resolution.

If we want to introduce a time resolution of 1 μs and to keep the same maximum delay, the memory requirements of the PSP buffers increase a thousand times. Moreover, increasing the time resolution means that the periodic update process will be executed more often and thus will have less time to update the state of the population. Going from a millisecond to a microsecond resolution will reduce the time available for state update by a factor of 1000.

It is possible to consider these trade-offs but this results in an dramatic decrease of the number of neurons which can be simulated on a core, going from 100 neurons to only a handful of neurons. Implementing huge neural networks with microseconds resolution is thus not practical with the current available implementation.

## 3. Going beyond the millisecond

This section describes the changes to the standard SpiNNaker software package we implemented to allow simulation with microsecond precision. The changes to the base tools are first discussed, then new neural models are introduced.

### 3.1. Tools and support

#### 3.1.1. SpiNNaker API

Applications written for the SpiNNaker cores rely on an Application Programming Interface (API) provided by SpiNNaker's builders. This API provides a set of support functions and a framework allowing the use of the different resources offered by the hardware platform.

Firstly, measuring time with microsecond resolution had to be added to the API which normally measures time with a resolution corresponding to the timestep of the simulation (1 ms by default). This can be easily implemented by using the more precise value of the hardware counter already used by the API to deduce the number of microseconds which have elapsed since the last simulation timestep. This allows precise time measurements without an extra cost in resources.

Secondly, all SpiNNaker events related to timings are based on the simulation clock and thus inherit the timestep resolution. To solve this problem, we use the second hardware timer available in the SpiNNaker cores to generate interrupts with a user settable number of microseconds delay. This allows the scheduling of events which will be triggered with microsecond resolution using the existing framework.

#### 3.1.2. Monitoring spikes

To record spikes from a neural simulation in SpiNNaker, a monitoring core runs a special application which collects spikes from the populations selected for recording by the user and sends them to a host computer, in charge of collecting simulation results, while the simulation is running. This is done using a special type of packet which can carry a payload of 256 bytes.

The current implementation of this monitoring application sends a packet of spikes every millisecond. The timestamps of the spikes are indicated in the header of the packet which can then carry a total of 64 spikes. The same scheme cannot be used for microsecond resolution: it would require sending up to a packet every microsecond, potentially containing only one spike. This would dramatically increase the overhead due to the packet headers and would require the emission of too many packets.

To overcome this problem, we implemented a new monitoring application. This core now stores incoming events as a pair of values: (key, timestamp). The key corresponds to the ID of the neuron which spiked and the timestamp indicates the time, in microseconds, when this spike has been received. These pairs are stored in buffers of 256 bytes (32 spikes), which are sent to the shared SDRAM of the chip when full, using DMA. Another process then reads these buffers from SDRAM and sends packet containing the events to the host computer when needed.

This allows more events to be recorded per millisecond than the previous implementation, while allowing microsecond resolution in their time of arrival. Moreover, the events can be stored in SDRAM quicker than they are sent to the host computer, allowing to increase the total amount of data which can be acquired during the simulation (The user will just have to wait for the data to be completely transferred to the host computer after the end of the simulation). This scheme of pairing addresses and timestamps is also used in other programs such as the jAER program (jAER, 2007) and thus facilitates integration with such tools for real-time software implementation.

#### 3.1.3. Generating input spikes

A dedicated application exists to generate input spike trains which appear to come for another population of neurons. The application provided in the standard SpiNNaker implementation uses a particular format to store this information. Spikes are stored in bitmaps. For every simulation timestep, a chunk of memory is read. In this memory portion, each bit codes for one neuron of the source population. If the bit is set to 1, this particular neuron has to produce a spike in the current timestep. If the bit is set to 0, it has to remain silent.

Considering the usual sparseness of spike data, it would be really inefficient to use the same scheme with microsecond resolution. This would require an enormous amount of memory directly proportional to the number of microseconds in the simulation. To improve this process, we implemented a new spike source application. This application represents spikes as pairs of values: (ID, timestamp). The information is stored in SDRAM by the host computer before the start of the simulation and read via DMA throughout the simulation. When a spike is produced, the application reads the time when the next spike should be produced. This allows us to compute the wait time before producing this next spike. An event is then scheduled in the API to happen after this delay. This process goes on during the whole simulation time until the end of the spike data. A pipelined process using a double buffer technique allows one to read the spike data from memory using DMA without adding delays in the replay process sending them to the other neural models of the simulation.

### 3.2. Neural models

#### 3.2.1. Dendritic delays

As we can see from the standard implementation provided with SpiNNaker, managing synaptic delays when simulating neurons can lead to an important memory usage. To solve this problem, we chose to implement these delays independently from the rest of the neural simulation.

One dendritic delay core implements one particular delay value. When a packet containing a spike is received, it is stored in a ring buffer in DTCM (local memory of the core). Then, a second process schedules events to dispatch these spikes after the given delay of the core has elapsed. This allows very compact and efficient code because events are output in their order of arrival.

Because there are a large number of cores available in a typical SpiNNaker machine, using one core per delay value is not troublesome. Moreover, one could configure a network where a spike goes several times through the same delay core to implement multiples of a base delay: if one core implements a delay of 100 μs, it can be used to realize a delay of 300 μs by routing events three times through the core before delivering the spike to its target neuron.

#### 3.2.2. Synchrony detectors

Detecting temporal coincidence between two spikes is a widely used feature in spiking neural networks (Carr and Konishi, 1988; Coath et al., 2013). As a consequence we decided to implement a dedicated core for this task instead of using standard integrate and fire neurons which would introduce an unnecessary overhead.

Each neuron simulated by this core has two types of synaptic input and a time window. When an incoming spike is received on one input, the core will output a spike if another spike was received on its second input in the given time window. We added a refractory period to this process to limit the maximum firing rate of the neurons if required.

#### 3.2.3. Leaky integrate and fire neurons

While an *ad hoc* solution can be introduced in the case of synchrony detection, for the rest of the network simulation we need to simulate standard neural models. This is done through a new core which implements leaky integrate and fire models with exponential kernels. These neurons are completely event-driven and have microsecond resolution.

As in the standard models, incoming packets carrying input spikes are received and queued in memory for processing (this process has to be done as soon as possible to ensure no back-pressure signal is propagated to the SpiNNaker router by not reading packets, as described in the next section). In addition to this standard processing, we also timestamp the spike on arrival to be sure to it is processed correctly even if events are queued for a variable amount of time. When such a spike is processed, a DMA call is issued to get the associated synaptic information from SDRAM.

Upon receiving the synaptic data from memory, the membrane potential of the post-synaptic neuron is updated with the exponential kernel and the spike's contribution is added to this potential. Its value is then tested for an output spike which is immediately produced if necessary.

It is worth noting that an output spike can only be produced at the time of an input spike which is compatible with an exponential kernel and with the absence of synapse dynamics (the contribution of a spike is an instantaneous addition/subtraction to the membrane potential according to the synaptic weight).

To optimize the process, the exponential kernels are precomputed by the core before the beginning of the simulation via two look-up tables. When we update the membrane potential, we need to compute the kernel decay over the time Δ_*t*_ which has elapsed from its last update:

(1) $$e^{-\Delta_{t}/\tau }=e^{-(\Delta_{t}^{\text{ms}}+\Delta_{t}^{\mu \text{s}})/\tau }=e^{-\Delta_{t}^{\text{ms}}/\tau }e^{-\Delta_{t}^{\mu \text{s}}/\tau },$$

where Δ^ms^_*t*_ is the maximum multiple of milliseconds contained in Δ_*t*_, Δ^μs^_*t*_ is the remaining number of microseconds and τ is the time constant of the neuron. To reduce the memory footprint required by the look-up table, we compute one table with microsecond resolution for the *e*^−Δ^μs^_*t*_^/τ part over a timespan of 1 ms and a second one with millisecond resolution for the *e*^−Δ^ms^_*t*_^/τ part over a timespan which can be configured in the code depending on the available local memory and time constant τ.

#### 3.2.4. Plasticity

Implementing plasticity on the SpiNNaker system is not a trivial task, due to its peculiar set of constraints and architectural characteristics. Rules that rely on spike timing can be triggered by the arrival of a pre-synaptic spike, inducing depotentiation, or the emission of a post-synaptic spike signaling depotentiation. This is for instance the case for spike timing-dependent plasticity (STDP) (Bi and Poo, 1998). On SpiNNaker, weights from SDRAM are only available in the local memory of an ARM core upon the receipt of a MC packet. This triggers a lookup in memory fetching all the weights associated with the incoming spike. Weights are therefore available in memory only when a packet is received. Due to the nature of how delays are normally implemented on the SpiNNaker architecture (see Section 2.3), this time does not correspond to the time the spike arrives at the post-synaptic neuron, as the delay is reintroduced post-synaptically. Moreover, when a post-synaptic spike is produced weights are in SDRAM, with no local pointer to them; retrieving them selectively would be difficult as they are indexed by pre-synaptic neuron, hence scattered when considering post, leading to unoptimized memory transfers.

The deferred event-drive model (DED) (Jin et al., 2009) was introduced to circumvent these problems. The approach consists in gathering information about spike timing and deferring plasticity into the future, once all the information required is available. In our implementation, we decided to use the voltage-gated STDP variant introduced by Brader et al. (2007). This rule is particularly appealing for neuromorphic implementation (see for example Mitra et al., 2009) because it is only triggered on the arrival of a pre-synaptic spike, solving the first of the two problems associated with plasticity implementation. Our novel delay cores take care of solving the second problem, the reintroduction of the delay at the post-synaptic end. By using these cores, the time of arrival of a MC packet corresponds to the time when a spike needs to be computed at the post-synaptic end. This rule depends on a post-synaptic trace *C*(*t*) representing the calcium concentration and which evolves accordingly to the firing activity of the neuron:

(2) $$\frac{dC(t)}{dt}=-\frac{C(t)}{\tau_{C}}+J_{C}\sum_{i}\delta (t-t_{i})\;\;,$$

where *t*_*i*_ are the post-synaptic spike times. *C*(*t*) triggers potentiation and depression as follows: if *C*(*t*) is in an interval [θ^*h*^_*down*_, θ^*h*^_*up*_] potentiation is triggered; otherwise if *C*(*t*) is between [θ^*l*^_*down*_, θ^*l*^_*up*_] depression is triggered. This variable is computed using an exponential LUT in a similar way as done with the membrane potential. The plasticity rule depends also on the post-synaptic membrane depolarization *V*(*t*) according to a threshold value θ_V_, sampled at the time of arrival of a pre-synaptic spike (*t*_*pre*_). Weight dynamics *w*(*t*) can then be summarized as follows:

(3) $$w=w+a\text{ if }V(t_{pre})>\theta_{V}\;\;\;\text{ \&}\;\;\;\theta_{down}^{h}\leq C(t_{pre})<\theta_{up}^{h}$$

(4) $$w=w-b\text{ if }V(t_{pre})\leq \theta_{V}\;\;\;\text{ \&}\;\;\;\theta_{down}^{l}\leq C(t_{pre})<\theta_{up}^{l},$$

where *a* and *b* represent the constant weight increase and decrease values, respectively.

The plasticity rule provides relaxation toward two stable states, if none of the conditions in Equation (3, 4) are fulfilled. The weight *w* drifts linearly with rate α toward *w*_*max*_ if its value is greater than a threshold θ_*W*_; conversely it drifts linearly toward *w*_*min*_ with rate β leading to the additional dynamics:

(5) $$\frac{dw(t)}{dt}\;=\alpha \text{ if }w(t)>\theta_{W}$$

(6) $$\frac{dw(t)}{dt}\;=-\beta \text{ if }w(t)\leq \theta_{W}$$

Using this system we can efficiently compute weight updates upon the arrival of a pre-synaptic spike, as all the information required by the algorithm are locally available in the neural core.

## 4. Results

This section presents the time characterization of the platform and of the novel software infrastructure introduced. The section concludes by showing two simple experiments which use the new features: a binaural model for sound localization and a plastic model capable of learning precise temporal relationships in spike trains.

### 4.1. Intra- and inter-chip MC packet latencies

The MC router is responsible for communicating the spikes to its internal cores or to other chips through six asynchronous bi-directional links. Its pipelined implementation enables it to route one packet per clock cycle to all or a desired number of output links in an uncongested network. If any of the output links is busy, the router will retry to route the packet at every clock cycle until it reaches a predefined number of clock cycles after which it will attempt emergency routing via the link which is rotated one link clockwise from the blocked link (not applicable for destinations internal to a SpiNNaker chip). Similarly, if the emergency route fails the router will retry emergency routing at every clock cycle until it reaches a second user-defined number of cycles when it finally drops the packet.

This section describes a series of experiments conducted in order to investigate the intra- and inter-chip MC packet latencies as a function of the router's waiting time and synthetic traffic going through a link. For these experiments, a parameterized software was developed using the SpiNNaker API. The packet received callback priorities were set to minus one (pre-emptive) in order to ensure that packets were cleared from the communications controller immediately upon receipt. The timer tick callback priority, which was used by the cores for terminating the simulation after 60 s, was set to zero (non-queueable priority). Finally, the priority of the callback function developed to generate MC packets was set to two (lowest queueable priority). The processor clocks were set to 200 MHz, routers and system buses to 100 MHz, while the off-die memory clocks to 133 MHz.

#### 4.1.1. Intra-chip MC packet latency

**Figure 3:**
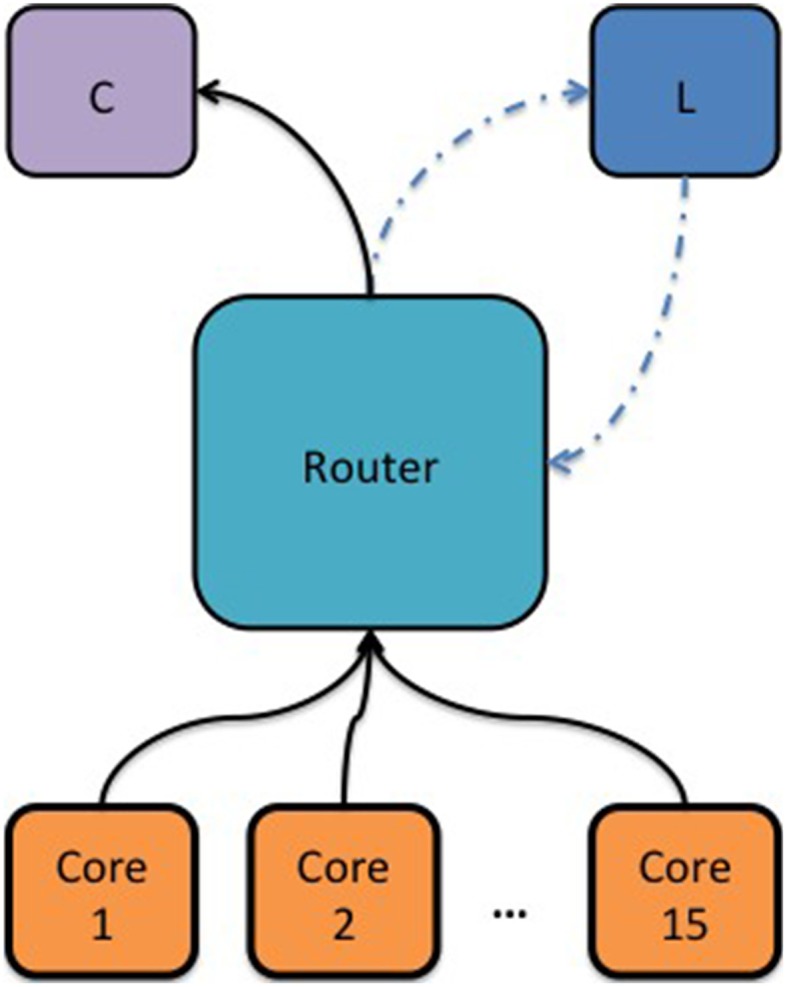
**Block diagram showing the topology used to measure the intra-chip packet latency as a function of a congested link and the router's waiting time before dropping a packet**. Cores 1–15 were used to generate synthetic MC packets and the router would redirect these packets to a consumer core (C). An additional core was used to measure the MC packet latency (L) by sending a packet to the router periodically and receiving it back. A hardware timer was used to measure the time passed from sending a MC packet to receiving it back.

The first experiment was aimed at demonstrating the core-to-core packet latency within a SpiNNaker chip as a function of a congested internal link and the wait time of the router before dropping a packet. Congested link means that a core has received more packets than it can process on time and a back-pressure signal will propagate to the router through that link. For this experiment, 17 cores were used. One core was dedicated to measuring the core-to-core MC packet latency within a SpiNNaker chip. By sending a packet to the router every 500 ms using the “timer tick” callback function and receiving it back. The second hardware timer, available on each core, was used to count the clock cycles between sending and receiving the MC packet (with nanosecond resolution). Each of the remaining 15 cores would generate approximately 1.7 million packets per second, which would be routed to one particular consumer core (C) whose sole task was to count the received packets, Figure 3. The logged MC packet latencies during the simulation, the values of the software counters and additional diagnostic information from the router were uploaded to the SDRAM when the simulation was over and fetched by the host for further analysis.

**Figure 4:**
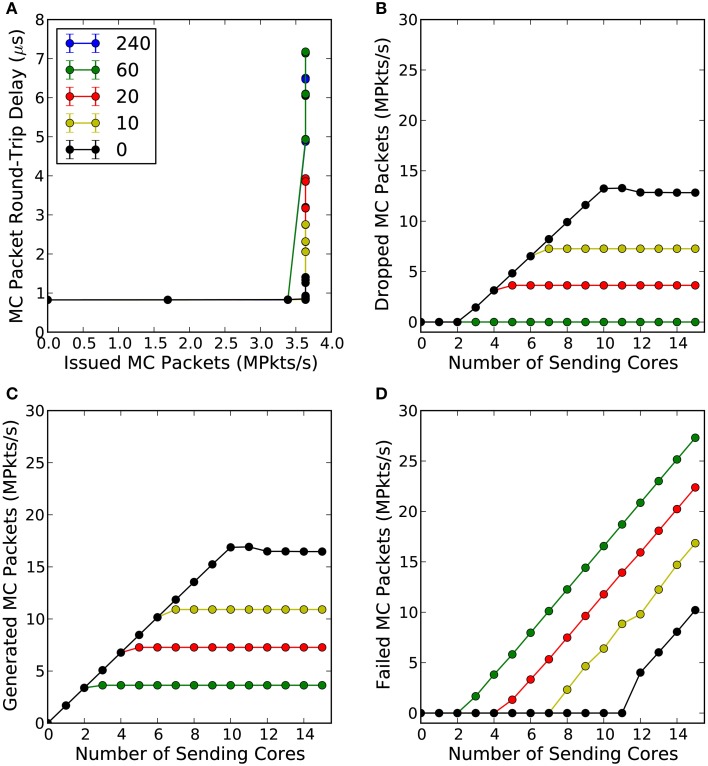
**(A)** Shows the mean intra-chip MC packet round-trip delay time as a function of the total number of packets per second the router issued to the consumer core (C), for different router wait times (default value is 240 cycles). **(B)** Shows the total number of packets per second the router dropped as a function of the number of cores participating in the simulation and for different router wait times. **(C)** Shows the total number of packets per second that were successfully generated by the communication controller as a function of the number of cores participating in the experiment and for different wait times. Finally, **(D)** shows the total number of packets per second the communications controller was not able to send to the router due to the back-pressure of a congested link, for different wait times. Each core attempted to inject 1.7 million packets per second to the consumer core through the router.

Figure 4 shows the mean and standard deviation of the intra-chip MC packet round-trip delay time (RTD) as a function of the total number of MC packets per second the router has issued to the consumer core (C) and for various router wait times. What can be observed from this figure is that in an uncongested network, the intra-chip RTD time is constant at 0.825 μs, Figure 4A. Within this time is included the software overhead of the SpiNNaker API required to write the MC packet to the communication controller, the time needed for the packet to traverse through the internal link to the router, the time required for the packet to go through the router and again through the internal link to the communication controller of the target core and finally the software overhead of receiving the packet from the communication controller. The aforementioned times can be expressed as:

(7) $$t_{\text{RTD}}^{\text{Intra}}=t_{\text{Send}}^{SW}+2\;\cdot \;t_{\text{Link}}^{\text{local}}+t_{R}+t_{\text{Receive}}^{SW}\;\;,$$

where *t*^*SW*^_Send_ is the software overhead of the API required to write the key of a MC packet to the communication controller, *t*^*SW*^_Receive_ is the time passed from handling the interrupt raised by the communication controller to branching to the callback function assigned to handle the packet received events, *t_R_* is the time required by the router to process a single MC packet, and finally *t*^local^_Link_ is the time a single MC packet needs to go through the local links.

The mean *t*^*SW*^_Send_ and *t*^*SW*^_Receive_ times (averaged over 4 trials) were found to be 0.415 μs and 0.13 μs, respectively, by utilizing the second hardware timer. Assuming that the time consumed by a MC packet to traverse through the local links is much smaller than the time spent by the router to process a packet, the *t*^local^_Link_ can be ignored. Solving Equation 7 for *t_R_*, the time the router requires to process a single MC packet is 0.28 μs.

As soon as congestion occurs, which for this experiment happens when the consumer core (C) receives more than 3.6 million packets per second, the communication controller of the consumer core (C) starts adding back-pressure on the router, which attempts to resend the packet at every clock cycle until it reaches a predefined number of cycles (240 default) at which point the packet is finally dropped, Figure 4B. This back-pressure signal propagates back along the pipeline and the router stops receiving new packets until back pressure has been released (Wu and Furber, 2010). As a consequence, the MC packet latency increases and the hardware buffers of the communication controllers of the cores generating the MC packets are not emptied; this explains why the total number of generated packets plateaus, as seen in Figure 4C, while failed packets increase (software buffer full), see Figure 4D. For the router's default waiting time (240 cycles) and for waiting 60 cycles no packets were dropped in any of the trials but the worst-case RTD time went up to 6.5 μs. For router wait times of 20 cycles and below, the worst-case RTD time drops below 4 μs but the total number of dropped packets per second increases dramatically. This trade-off between intra-chip MC packet latency, packets being dropped or not being sent to the router at all, requires further investigation as it depends on the requirements of a particular application.

#### 4.1.2. Inter-chip MC packet latency

**Figure 5:**
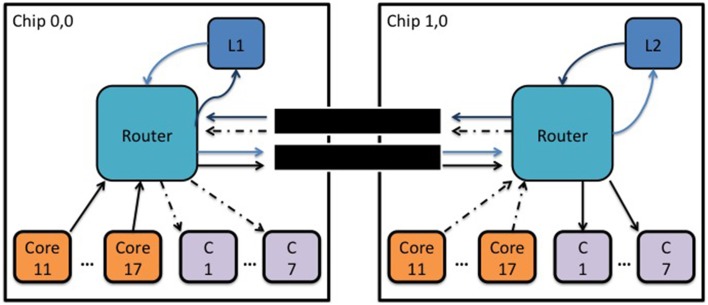
**Block diagram showing the topology used to measure the inter-chip MC packet latency as a function of synthetic traffic going through a link**.

The router of a SpiNNaker chip can communicate packets to neighboring chips through six self-timed bi-directional links. The average bandwidth (transmit/receive) of each link is 6 million packets per second (240 gigabits per second) and this may vary with the temperature, voltage or silicon properties. An experiment was conducted to determine the inter-chip RTD of a MC packet transmitted through one of the 6 bi-directional links as a function of the link's outgoing and incoming traffic. For this experiment a core (L1) generates a MC packet every 500 ms and the router routes it to a neighboring chip through one of the six bi-directional self-timed links. Upon receiving the packet the second router would route it to a particular core (L2), whose sole task was to change the key of the packet and retransmit it back to the router which had an appropriate routing entry to route it back to the originating core, Figure 5.

Seven cores on each chip were used to generate packets which were routed to seven consumer cores (C) on the adjacent chip following a one-to-one mapping. This way the total number of packets per second a consumer core receives remains below 3.6 million packets per second, (which is the maximum number of packets a core can receive) ensuring that no additional pressure is added to the routers.

The generated packets were controlled by an inter-packet interval (IPI) parameter, which is a delay in microseconds before transmitting the next MC packet. Results are presented as the percentage of the utilization of the incoming and outgoing packets going through a link per second, with 100% utilization meaning 6 million packets per second.

**Figure 6:**
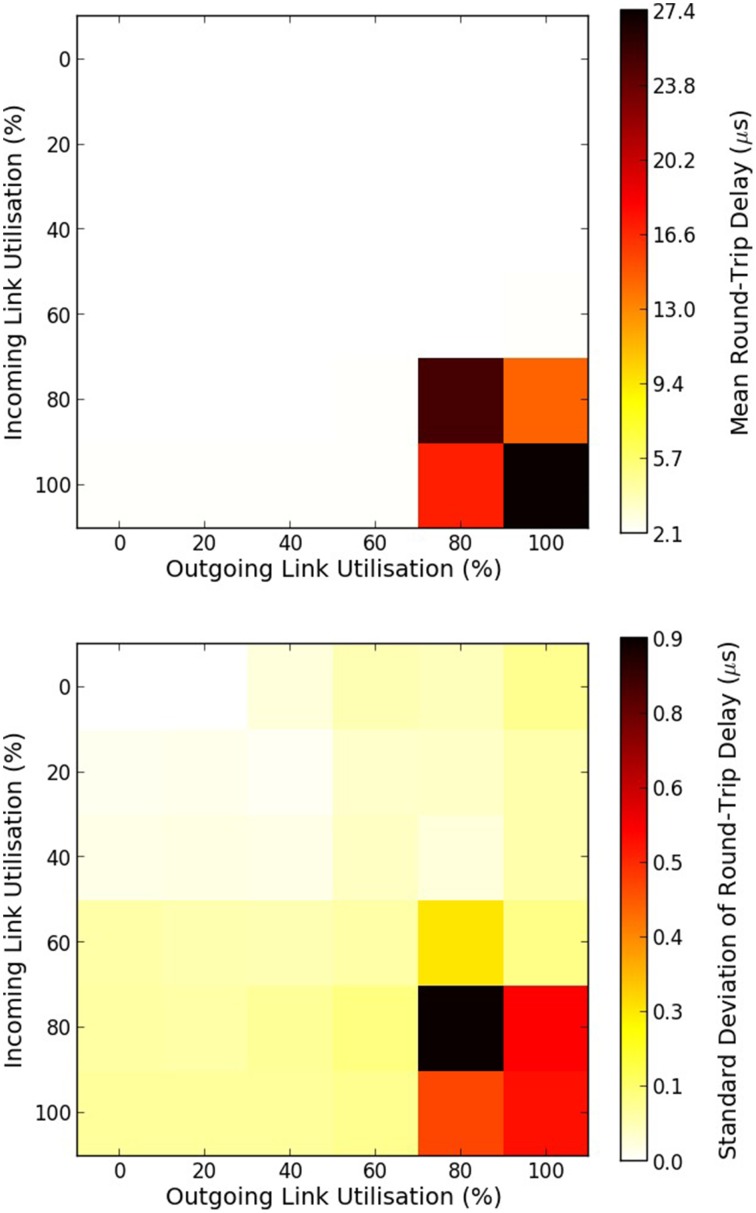
**Mean and standard deviation of round-trip delay times of MC packets as a function of the percentage of a link utilization**. Top figure shows the mean round-trip delay time, while bottom figure shows the standard deviation in microseconds. 100% utilization means 6 million outgoing/incoming packets per seconds going through a bi-directional link.

The mean and standard deviation of the RTD times of MC packets are presented in Figure 6. When there is no traffic the RTD time of a MC packet is 2.535 μs. Within this time is embedded the time required for the packet to go through each router twice, twice through the external link, and also two software processing overheads of sending and receiving the packet back to the router. This can be expressed as:

(8) $$\begin{array}{l} t_{\text{RTD}}^{\text{Inter}}=2\;\cdot \;t_{\text{Send}}^{SW}+4\;\cdot \;t_{\text{Link}}^{\text{local}}+4\;\cdot \;t_{R}\\ \;+2\;\cdot \;t_{\text{Receive}}^{SW}+2\;\cdot \;t_{\text{Link}}^{\text{external}} \end{array}$$

where *t*^external^_Link_ is the time a MC packet requires to traverse through an external link to a neighboring chip. Solving for an RTD time of 2.535 μs and by using the results of Equation (7) for *t*_*R*_ and by ignoring the time of *t*^local^_Link_, the time a MC packet needs to go through an external bi-directional link is 0.1625 μs.

For a link utilization of 60%, for both outgoing and incoming traffic, there is a 3% increase in the RTD time. When both the incoming and outgoing link utilization reaches 80% a very small number of packets were dropped as both routers attempted to reroute the packets for the default wait times (240 cycles), hence the dramatic increase in the RTD times. Results are summarized in Table 1.

**Table 1:** **Experimental results of the SpiNNaker latencies**.

**Parameters**	**Values**	**Units**
*t*^SW^_Send_	0.415	μs
*t*^SW^_Receive_	0.13	μs
*t*_*R*_	0.28	μs
*t*^external^_Link_	0.1625	μs

### 4.2. Time characterization

To test latencies in the system we build and simulate a very simple network composed of three populations:

- a spike source, producing spikes with microsecond resolution,
- a dendritic delay population which delays spikes with a tunable delay,
- a population of integrate and fire neurons in which synaptic plasticity is enabled or not in the implementation.

We add to the global architecture the monitoring application which records spikes from these 3 populations. All these applications run on the same SpiNNaker chip. From here, we can characterize timings and latencies by either looking at timestamps generated by the monitoring core which gives us a common clock for all of the activity on the chip or by keeping track of the timings directly in the different cores by adding debug instructions to their processing.

We start by characterizing the spike source application. By adding debug instructions in the code, we can output the time at which spikes would be sent without this debugging overhead and compare them to what is asked from the core. Our measurements show that every spike is reliably emitted with a 2 μs delay after the timestamp set in the simulation script in conditions where no delays are introduced by congestion due to too much activity in the chip. Looking at the same events in the output of the monitoring application, we can see that they are consistently timestamped with a 3 μs. The 1 μs difference between the two numbers is due to the overhead of the API when sending a spike, that is, the time it needs to travel from one core of the chip to another one (which has been characterized in the previous subsection) and the time needed by the receiving core to timestamp the spike. From the results of the previous subsection (4.1.1), we can consider than under normal operational conditions (no excessive load of events in the system), this time will be constant for every population on the same chip because they use the same code to send packets to the system. Thus, from now on, we can use the timestamps from the monitoring application to know when spikes are emitted by each core recorded during our simulations.

We then look at the dendritic delay population. This population directly receives input from the spike source and the delayed output is recorded by the monitoring application. By comparing the timestamps of the spikes received from the spike source and the ones received from the dendritic delay core, we can compute the actual delay introduced by this population. By removing its set delay, we obtain the overhead introduced by the implementation. For different delays and input spikes, we reliably get an overhead of 2 μs which can be compensated when specifying a desired delay in a simulation.

This delay population is then connected to a population of integrate and fire neurons implementing plasticity as described in the previous section. The connection between these two populations is an all-to-all connection. This allows us to vary the size of the synaptic data fetched from memory, which directly depends on the number of post-synaptic neurons associated to each pre-synaptic one. This enables us to vary the amount of processing each incoming spike requires (more post-synaptic neurons means more neurons to update when receiving a spike), thus allowing easy characterization of the following latencies:

- the initial latency, corresponding to the time required to receive the spike and fetch the synaptic data from memory. This time has been measured to be 4 μs,
- the time required to update each post-synaptic neuron targeted by the incoming spike. This time has been measured to be 1.6 μs,
- the time required to send a spike which has been measured to be 0.4 μs.

These timings allow to compute the required update time *T*_*u*_ required by a spike for a given network topology with the following formula:

(9) $$T_{u}=4+2\;\cdot \;N_{\text{max}}^{\text{post}}\;\mu \text{s},$$

in the worst case scenario where every pre-synaptic spike produce a spike in each one of its post-synaptic neurons and where *N*^post^_max_ is the maximum number of post-synaptic neurons a pre-synaptic one connects to.

If we remove the plasticity computation from the model, the initial latency and the time needed to produce a spike stay the same. The only modification can be found, as expected, in the time needed to process a spike which drops to 0.5 μs.

### 4.3. Detecting sub-millisecond spike synchrony in a model of sound localization

**Figure 7:**
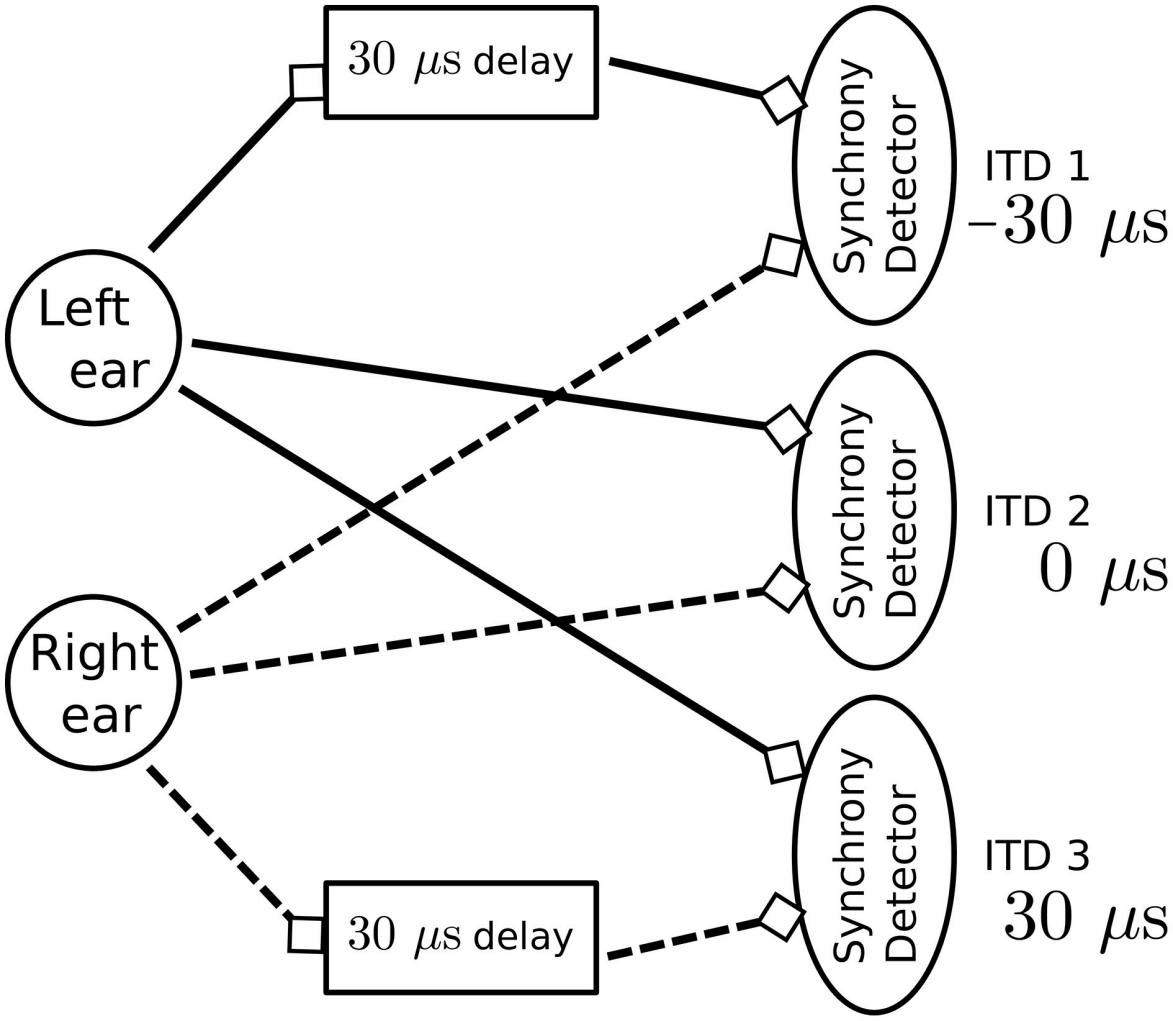
**Model used to detect sub-millisecond spike synchrony for sound localization**. The model consists of three synchrony detectors where each is activated by a different input interaural time difference (ITD). To achieve this result, each detector is directly connected to one ear whereas the second input comes from a dendritic delay population. For positive ITDs, spikes from the right ear are delayed. For negative ITDs, spikes from the left ear are delayed. In this experiment, spike trains from the two ears are simulated for three sound sources localized at positions producing expected ITDs.

**Figure 8:**
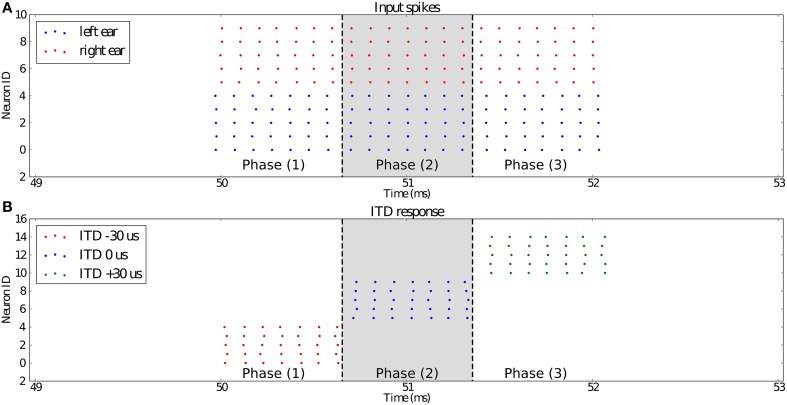
**(A)** Shows the input spikes to the system. It is comprised of the spikes (5 channels, red dots) from the right ear and the spikes (5 channels, blue dots) from the left ear. They correspond to a sound source positioned at ITD −30 μs for the first third [phase (1)] of the input stimulus, then ITD 0 μs for the second third [phase (2)], and 30 μs for the last part [phase (3)]. **(B)** presents the outputs of the three synchrony detectors of the network configured to respond to ITDs −30 μs (red), 0 μs (blue), and 30 μs (green). We can see that for each channel, the detector with its preset ITD fired correctly in each phase of the experiment.

To test the dendritic delay and synchrony detector models, we simulate a standard network used for sound localization. This model is presented in Figure 7 and results are presented in Figure 8. For each ear, we consider a population of neurons representing 10 different frequency channels (Figure 8A). We start by generating spike trains with an interspike interval (ISI) of 100 μs for each of these channels and we feed them in the right ear (red dots). Then, this simulated sound is shifted in time according to the input interaural time differences (ITDs) corresponding to values compatible with human hearing: -30 [phase (1)], 0 [phase (2)] and 30 μs [phase (3)] to generate the input spikes for the left ear (blue dots). Some noise is then added independently to spikes from each ear and each channel by jittering each spike randomly between -5 and 5 μs to get the actual input presented in Figure 8A. Each ear is then input in delay lines and synchrony detectors such as to detect the corresponding ITDs, synchrony detectors are, because of their associated delay lines, centered around -30, 0, and 30 μs with a window of 15 μs. These detectors are color coded in Figure 8B with detectors for ITDs -30, 0, and 30 μs, respectively corresponding to red, blue, and green dots. We can see that the different input ITDs are correctly extracted by the architecture for each phase of the input pattern.

### 4.4. Learning temporal patterns with sub-millisecond precision

**Figure 9:**
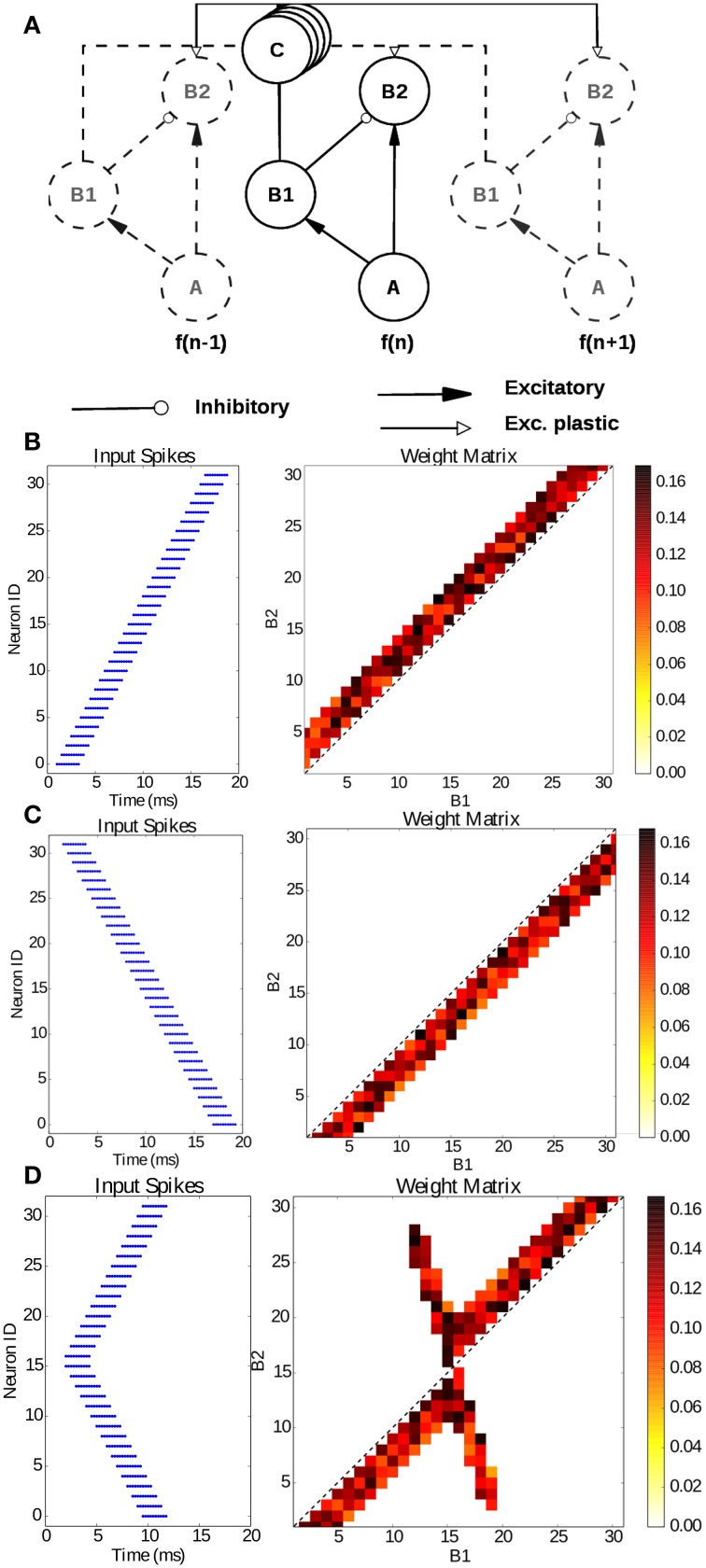
**Learning sub-millisecond time patterns. (A)** Network structure: input spikes are produced by neurons A, which represent frequency channels of a simulated silicon cochlea; they drive neurons B1 and B2 with excitatory synapses; B1 is connected to the B2 neuron of its own channel with an inhibitory synapse, while it is connected to the other channels through the delay populations C with plastic connections. Delays are proportional to the tonotopic distance between channels. **(B)** Input raster plot (left) and final weight distribution (right) when stimulating the network with a forward frequency sweep, activating all channels in rapid succession. **(C)** Input raster plot (left) and final weight distribution (right) when stimulating the network with a backward frequency sweep, activating all channels in rapid succession; **(D)** input raster plot (left) and final weight distribution (right) when stimulating the network with a forked frequency sweep starting from the central channels.

The model presented in the previous section describes how synchrony detection can be exploited through delay lines to localize the source of a sound. In this section, we use the model introduced by Coath et al. (2013) to learn spatio-temporal patterns with sub-millisecond precision. The model shown in Figure 9A, can be described as tonotopically organized channels, as it is in the auditory system. Each frequency channel consists of three neurons (*A*, *B*_1_, *B*_2_) interconnected through delay lines *C*. Within a channel, neuron *A*, representing inputs from a channel of a silicon cochlea for e.g., (Liu et al., 2014), connects to both neurons *B*_1_ and *B*_2_ through excitatory synapses while neuron *B*_1_ is connected to *B*_2_ through an inhibitory synapse and neuron *B*_2_ receives excitatory plastic input connections from the neighboring *B*_1_ neurons. These connections are mediated by the delay populations, implemented with the model described in Section 3, so as to have a delay proportional to the tonotopic distance between two channels. This delay line is the feature that enables learning of temporal patterns through coincidence detection.

To test our newly introduced SpiNNaker infrastructure for microsecond precision and delays, we reproduce the results published by Sheik et al. (2012) on the implementation of the model on a plastic neuromorphic analog VLSI multi-neuron chip. We present the same three spiking patterns as in the paper: a forward, a backward and a forked frequency sweep, activating all channels in rapid succession but with different time dynamics. Each channel is activated with 10 spikes with an ISI of 250 μ*s* and each channel is activated with a delay of 500 μ*s* after the previous channel. Each frequency sweep has been repeated 20 times, resulting in the connectivity matrices presented in Figures 9B-D which can be compared with Figures 7, 8 in Sheik et al. (2012). In each case training took approximately 4 s. The resulting weight matrices, initialized randomly, show how the network was able to learn a precise spatio-temporal pattern through coincidence detection. The learning process potentiates the synapses detecting the temporal features of the presented stimulus thanks to the tonotopic delays lines, converging in an emergent connectivity matrix which tunes the network to the presented stimulus.

The same experiment has also been replicated on SpiNNaker using the framework introduced in Galluppi et al. (2015), but with important differences in the temporal resolution and in the methodology to what is presented here. The temporal resolution of the former work is limited to the millisecond precision because of the structure of the neuron models and plasticity framework used. This new framework allows us to compute plasticity with a time resolution of less than a millisecond, which was not possible with the previous plasticity methods implemented on SpiNNaker.

When comparing our resulting weight matrices to the ones from Sheik et al. (2012), it can be noted that our results are not impacted by 2 important factors of the hardware system used in the original work: the precision of the synaptic weights and the mismatch between neurons. Since SpiNNaker is a digital platform, the precision of the weights can be changed depending on the application. As a result, our results show a finer scale in the weight matrix. Similarly, SpiNNaker neurons and synapses are not affected by hardware mismatch because of the digital implementation which results in less noisy weight metrics in our implementation.

This work matches the temporal resolution of the experiments in Sheik et al. (2012). Regarding the methodology, in this work we use a purely event-driven approach, that is plasticity is computed as soon as a spike is received, leveraging the simplification introduced by the separation of synaptic delays and neural models. This work hence constitutes the first implementation of plasticity on SpiNNaker where the weight update is not deferred into the future.

## 5. Discussion

In this paper, we showed that the current software package provided with the SpiNNaker platform was insufficient for certain applications requiring temporal resolution below a millisecond. To overcome this limitation, we introduced new software tools and models allowing to go beyond the millisecond barrier and reach microsecond precision.

To assert these new functionalities, we needed information about the timing involved at the level of SpiNNaker's fabric itself. We characterized the timing requirements of the hardware on one hand and of the software API on the other. This allowed us to fully characterize our implementation and provide insights about its computational limits for an user wanting to simulate a given network. We then demonstrated that our newly introduced architecture can simulate networks implementing sub-millisecond tasks and using plasticity by achieving, in real-time, sound localization, and sound patterns extraction with realistic spike trains.

It should be noted that these new tools do not change the way in which events are transferred in the global SpiNNaker architecture. They are just making the best of real-time to increase the time resolution of implemented models. Thus, they are fully compatible with the already existing models on SpiNNaker. This means that if a user wants to build a sensory processing neural network in which microsecond resolution is only needed in the early processing stages, he or she can use these microsecond precision models in these stages and then feed their output to standard neural models which will then compute with millisecond resolution for the later stages. This allows resources to be exploited maximally by tuning the time resolution to the requirements of the running model.

Notably we have introduced learning in our framework as it is a key process in developing precise coincident detectors. Furthermore, in the already mentioned owl auditory system, cross-modal interaction of different sensory systems appears to be crucial: visual cues guide the formation of a precise sound localization neural circuit (Gutfreund et al., 2002; Knudsen, 2002). These studies point at the importance of representing sensory inputs with high temporal resolution. In fact our newly introduced framework is much oriented to exploiting the enhanced temporal properties given by neuromorphic event-driven sensors (Liu and Delbruck, 2010), as silicon retinas (Lichtsteiner et al., 2008; Posch et al., 2011, 2014), and cochleas (Liu et al., 2014) which can seamlessly be interfaced with SpiNNaker (Galluppi et al., 2012a; Orchard et al., 2015). In this regard we have presented here a platform which offers a wide range of trade-offs in simulating spiking neural networks with different time-scales efficiently, and can be used for cross-modal learning.

### Conflict of interest statement

The authors declare that the research was conducted in the absence of any commercial or financial relationships that could be construed as a potential conflict of interest.
